# Could Breast Tomosynthesis With Synthetic View Mammography Aid Standard Two-Dimensional Mammography in Evaluation at Symptomatic Triple Assessment Breast Clinics?

**DOI:** 10.7759/cureus.18567

**Published:** 2021-10-07

**Authors:** Amy C O'Brien, Ailbhe O'Neill, Syer Ree Tee, Eileen Seymour, Sheena O'Keeffe, Sorcha McNally

**Affiliations:** 1 Department of Breast Radiology, St. Vincent’s University Hospital, Dublin, IRL; 2 Department of Radiology, St. Vincent’s University Hospital, Dublin, IRL; 3 Department of Medical Physics & Clinical Engineering, St. Vincent’s University Hospital, Dublin, IRL

**Keywords:** mammography, standard two-dimensional mammography, synthetic mammography, digital breast tomosynthesis, breast cancer, breast imaging, breast radiology

## Abstract

Background

This study aimed to determine whether breast tomosynthesis and synthetic view mammography (SM) can aid standard two-dimensional mammography (S2DM) in the evaluation of symptomatic women at triple assessment clinics (TACs).

Methodology

Digital breast tomosynthesis (DBT), SM, and S2DM were performed on 400 patients at symptomatic breast TACs between September 2020 and November 2020. Diagnostic findings on mammography and ultrasound were retrospectively recorded and analyzed by a breast-trained radiologist with 13 years of clinical experience. Pathology results for all biopsies were recorded and correlated with the mammographic and ultrasound findings.

Results

The combination of DBT and SM was superior to S2DM in the following settings: calcifications were more conspicuous on SM than S2DM in 44% of patients with calcifications. Mass margins were better defined on DBT than S2DM in 71% of patients with masses. Distortion was more easily detectable in 11% of patients with distortion on SM and in 44% of patients with distortion on DBT compared with S2DM. All malignant lesions were identified on all modalities.

Conclusions

Combined DBT and SM demonstrated several advantages over S2DM alone. SM can provide equal and sometimes superior diagnostic performance with the added benefit of requiring no additional radiation exposure when synthesized from DBT data. We conclude that adding DBT and SM to S2DM aids in the assessment of symptomatic women, and omitting S2DM results in no loss of clinically relevant information for women presenting to symptomatic breast clinics.

## Introduction

Digital breast tomosynthesis (DBT) was first approved by the Food and Drug Administration (FDA) in 2011 as a technique for breast imaging. Since then, multiple studies have demonstrated the usefulness of this modality in both the screening and diagnostic settings [1,2]. With DBT, mathematical algorithms are used to produce a volume rendering of the breast. Thus, overlapping breast tissues remain separated and the findings on various planes are more easily seen on individual slices [3]. Therefore, masses may be more conspicuous on DBT due to the effective removal of the superimposed tissue, resulting in better appreciation of the margins [4]. The inclusion of DBT with standard two-dimensional digital mammography (S2DM) improves cancer detection while simultaneously reducing the rate of false-positive examinations [5].

Despite this, the combination of DBT and S2DM has some disadvantages. One of which is the almost two-fold increase in radiation dose compared to S2DM alone [6,7]. In this study, the average glandular dose (AGD) was calculated for a subset of patients. The AGD for a single mammographic view was 2.3 mGy ± 1.0 (SD), and the range was 0.7-5.1 mGy for S2DM. The AGD was 2.7 mGy ± 1.0 (SD), and the range was 1.1-5.1 mGy for DBT. In our population, the mean unilateral DBT dose was 5.4 ± 2.0 (SD) mGy compared with the mean unilateral S2DM dose of 4.5 ± 1.9 (SD) mGy. Therefore, the combination dose of DBT and S2DM was nearly double that of S2DM or DBT alone.

Synthesized mammography (SM) is a technique in which a digital mammogram is generated from the DBT dataset and, therefore, does not require additional radiation exposure [6,7]. The AGD per view for a DBT examination, with synthesized views (SM), is typically higher (23-38%) than that for a standard digital mammogram alone [8]. With regards to the clinical performance of SM, there are reports that it may not be as effective as S2DM. Gur et al. demonstrated that a combined DBT and SM method has lower sensitivity for cancer detection than a combined DBT and S2DM method, which was mainly attributed to the poor quality of the SM images [7].

Because the back-projection algorithm used in the reconstruction of synthetic images is designed to preserve high-attenuating voxels, calcifications may appear enhanced [9]. Phantom studies have also shown that synthetic images have lower spatial resolution (or lower high-contrast spatial resolution) than two-dimensional (2D) digital mammography [10]. Despite this, early studies evaluating the performance of synthesized images have demonstrated no statistically significant difference in the detection of calcifications [11].

Advances have significantly improved SM image quality, and SM images are now approved for clinical use by the FDA, albeit not approved by all vendors, but only when interpreted in combination with DBT images [12]. Studies using improved SM images have reported that the diagnostic performance of these images is not inferior to that of S2DM images [6,13,14]. Nevertheless, there is concern that SM is inferior to S2DM as SM is not intended for stand-alone use in clinical practice, either for screening or diagnosis, and because it is only used as a complementary imaging modality along with DBT. These concerns have not been adequately addressed by clinical studies.

Therefore, we investigated the clinical use of DBT and SM in combination with S2DM imaging in a symptomatic population of women presenting to a triple assessment breast clinic (TAC) (including a physical examination, imaging, and biopsy if necessary).

## Materials and methods

Study population

In this institutional review board-approved retrospective study performed at a tertiary cancer center and a large academic hospital, the electronic radiology database was searched to identify patients presenting to symptomatic breast clinics between September 2020 and November 2020. These patients were referred by their general practitioners with breast masses on clinical examination, or other symptoms suspicious for breast cancer [15].

Inclusion criteria included consecutive patients who presented to the symptomatic breast clinic between September 2020 and November 2020 who underwent DBT with SM and S2DM. Exclusion criteria included patients with breast implants (these patients had implant included and implant displaced S2DM only without DBT), and patients who had S2DM performed within the last six months did not undergo repeat S2DM. During the data collection process, 415 patients were identified. Of these patients, 15 were excluded, including 11 patients with breast implants and four patients who had recently undergone imaging.

Data were collected retrospectively and stored using Microsoft Excel. Data collection was continued for the 400 patients who met the inclusion criteria. Additionally, pathology results were obtained and recorded retrospectively.

Mammographic technique

The 3Dimensions™ system (Hologic, Inc., Marlborough, MA, USA) was used in the ComboHD mode for the symptomatic breast on TAC patients (S2DM + three-dimensional (3D) tomosynthesis image set + synthesized 2D image) with single breast positioning and compression per view. S2DM alone, without DBT or SM, was performed on the asymptomatic breast. The synthetic view and Intelligent 2D™ software (Hologic, Inc., Marlborough, MA, USA) processes tomosynthesis data to generate 2D images which are designed to appear similar and serve the same goal as a digital 2D image (SM). Tomosynthesis images are acquired using an anode/filter combination W/Al along with automated exposure control and filtered back-projection (FBP) reconstruction combined with iterative calculation and 1 mm slice reconstruction. The S2DM is acquired using an anode filter combination W/Rd or W/Ag.

Image interpretation and data collection

A dedicated consultant breast radiologist participated in the observer study. The reader had six years of clinical experience reading DBT and 13 years of clinical experience reading S2DM. All images were interpreted on a dedicated digital mammography workstation (SecurView DX Workstation, Hologic Inc., Marlborough, MA, USA) equipped with a single 12-MP color LCD monitor (DWS-HLX-10.3.0.213; 4,200 × 2,800 pixels, 33.6 inches; Barco, Belgium). The reader individually analyzed the images in a standard mammography reading room. The S2DM, DBT, and SM images were all analyzed together and used in this study. The observer was blinded to patient names, ages, identification numbers, ultrasound findings, and pathology results to minimize learning bias.

The observer commented on mammographic findings, with morphological features of the detected lesion (mass, asymmetry, architectural distortion, microcalcifications) on S2DM, SM, and DBT; the probability of malignancy for any detected findings using Royal College of Radiologists (RCR) codes R2-R5 (R2 being benign findings, R3 indeterminate or probably benign findings, R4 suspicious of malignancy, and R5 highly suspicious of malignancy [16]); findings seen on SM or DBT alone; and findings appreciated more easily on DBT and SM compared to S2DM.

Statistical analysis

Assessments using S2DM and SM were divided into categories based on the R code and lesion morphology, as well as into benign or malignant categories based on histopathology results. Subgroup analysis was performed on patients grouped into masses, asymmetries, calcifications, and architectural distortion.

## Results

Mammographic findings and probability of malignancy based on imaging

In our study population of 400 patients, 30 (7.5%) had R5 lesions, nine (2.3%) had R4 lesions, 63 (15.7%) had R3 lesions, and 298 (74.5%) had R2 lesions. Biopsies were performed in 87 (21.7%) patients. This is a typical distribution of R codes and biopsies performed compared to the non-study population that present to our symptomatic breast TACs.

Of the 30 patients categorized as R5 (definitely malignant), all were malignant on histology. Of the 30 patients, masses were present in 20 (66.6%), calcifications in nine (30%), architectural distortion in seven (23.3%), and one (3.3%) patient had an asymmetry. Of the 30 patients categorized as R5, seven (4.3%) had an overlap of the above-mentioned imaging findings.

A total of nine patients had R4 (suspicious) lesions, of which seven (78%) were malignant on histology. Of the nine patients, two (22%) of the R4 lesions were benign, one of which was a fibroadenoma and the other was a papilloma on biopsy. In addition, four (44%) of the R4 lesions were calcifications, and five (56%) of the patients had masses on imaging.

A total of 63 patients had R3 (indeterminate) lesions, of which three (5%) were malignant. Atypia was seen in five (8%) patients with R3 lesions, 42 (66%) were benign, and 13 (21%) were not biopsied. The majority of R3 lesions not biopsied were skin lesions consistent with epidermal inclusion cysts, for which excision was suggested to the referring surgical teams. In total, seven (11%) of the R3 patients had calcifications, 47 (74%) of the patients had masses, and one (2%) patient had architectural distortion. The remaining eight (13%) patients had biopsies for clinical findings with no definite ultrasound or mammographic findings.

Overall in our study population, we had 40 (10%) malignant lesions, five lesions (1%) with atypia, and 13 (3%) lesions that required imaging follow-up (Table 1).

**Table 1:** Breakdown of outcome among 400 symptomatic patients who presented to the triple assessment breast clinic.

Population number	Benign	Requires short-interval imaging follow-up	Atypia	Malignant
N = 400	342 (86%)	13 (3%)	5 (1%)	40 (10%)

Digital breast tomosynthesis and synthesized mammography findings compared to standard two-dimensional digital mammography

**Figure 1:**
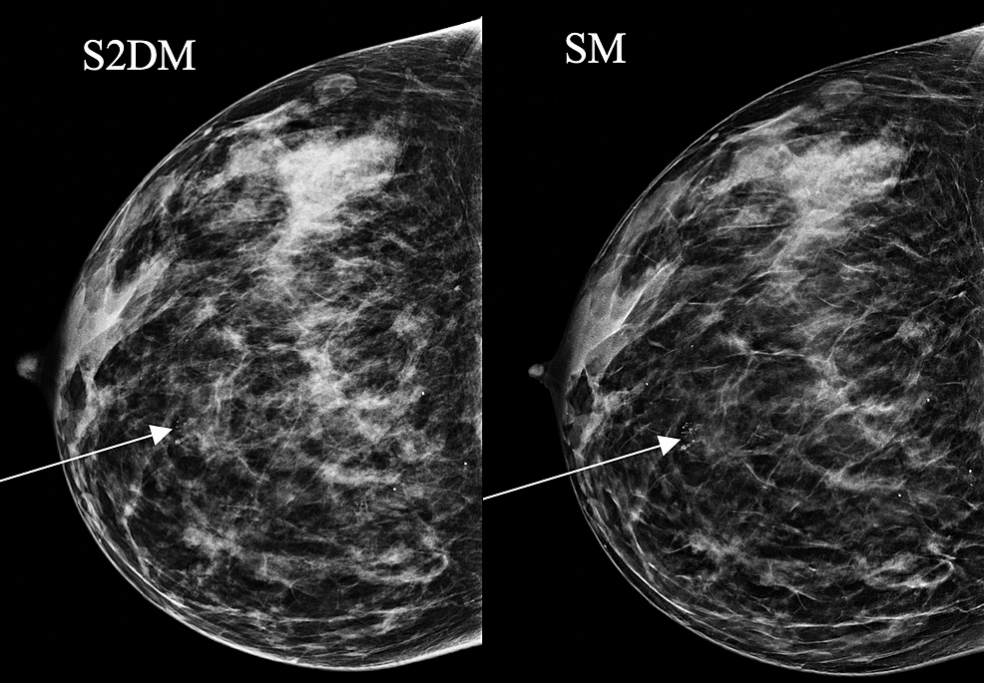
A 40-year-old woman who was breastfeeding presented with a right breast lump. Mammography showed calcifications more conspicuous on SM than S2DM (arrows). SM: synthesized mammography; S2DM: standard two-dimensional digital mammography

**Figure 2:**
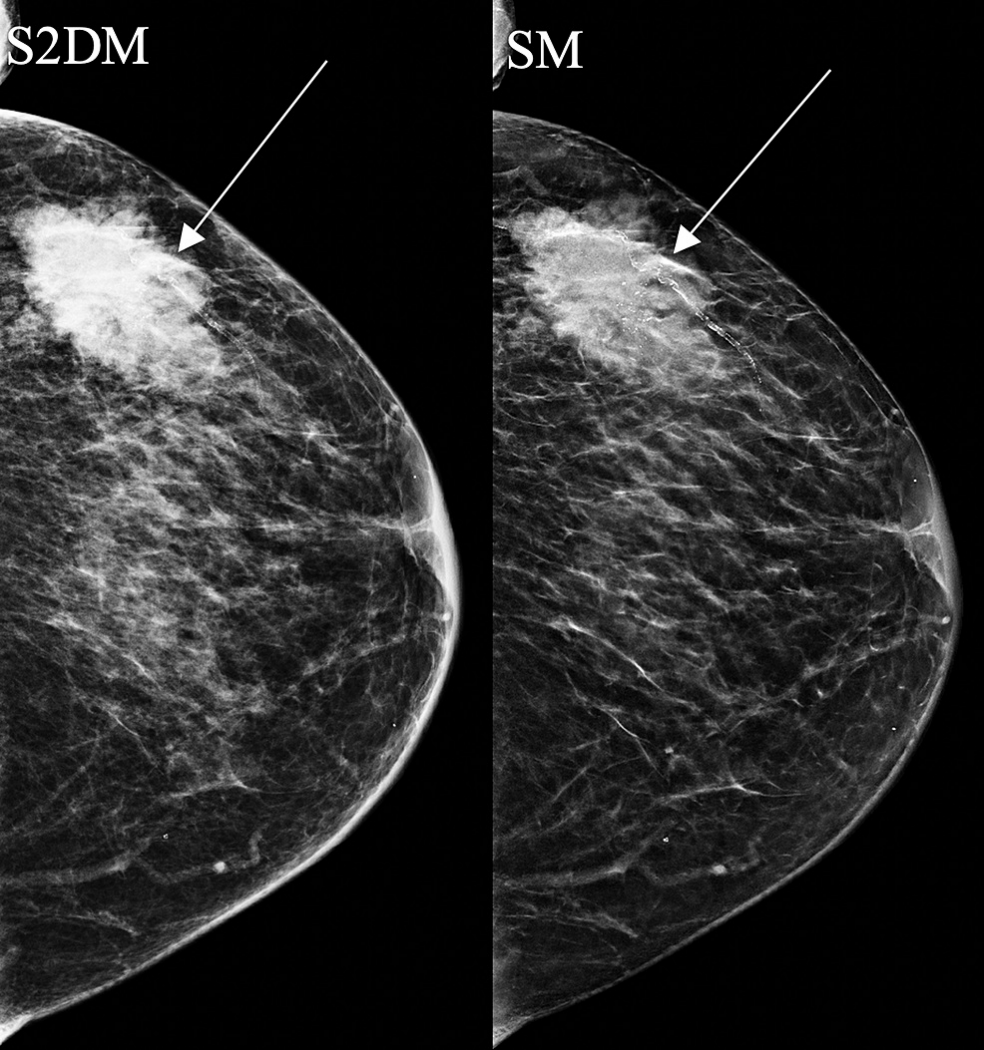
An 85-year-old woman with grade 3 invasive ductal carcinoma initially presented with a lump in the left upper outer breast. Mammography demonstrates a left breast mass with calcifications. The calcifications and spiculated mass margins are better defined on SM than S2DM (arrows). SM: synthesized mammography; S2DM: standard two-dimensional digital mammography

**Figure 3:**
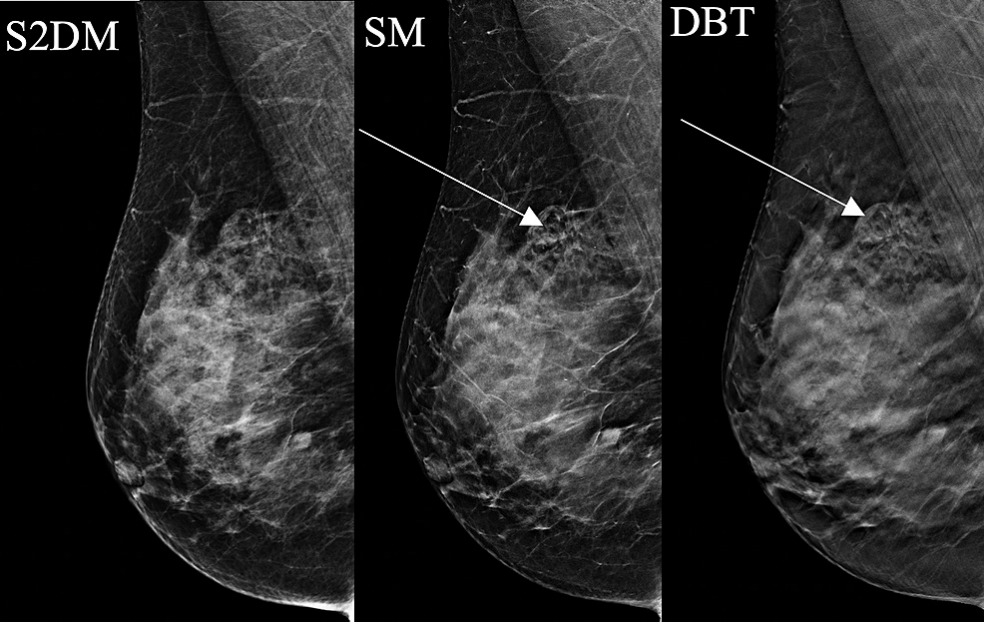
A 47-year-old woman presented with a right breast lump. Mammography demonstrates architectural distortion in the right breast. The distortion is only seen on SM/DBT (arrows). Biopsy confirmed benign breast tissue. SM: synthesized mammography; S2DM: standard two-dimensional digital mammography; DBT: digital breast tomosynthesis

All malignant lesions were identified on S2DM, SM, and DBT. However, SM was superior to S2DM in the following settings. Calcifications were more conspicuous on SM than S2DM in 17 (44%) out of 39 cases (Figure 1). Mass margins were better defined on SM than S2DM in nine (8%) out of 112 cases (Figure 2). Architectural distortion was more easily detectable on SM in one (11%) out of nine cases (Figure 3).

**Figure 4:**
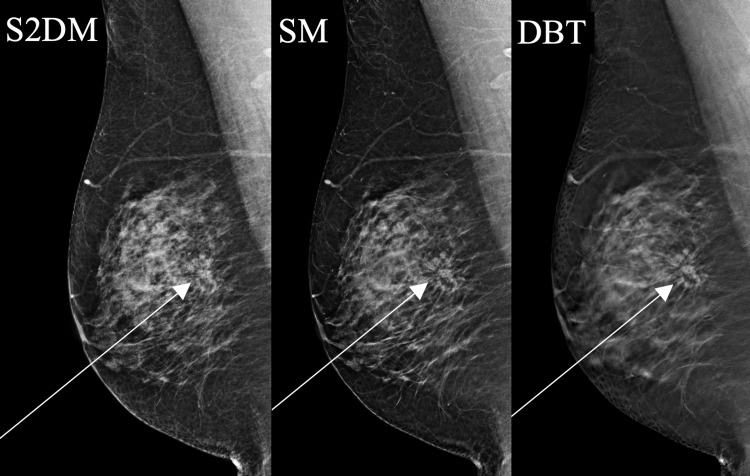
A 62-year-old woman with grade 3 invasive lobular carcinoma initially presented with a right breast lump. Mammography demonstrates architectural distortion which was easier to see on SM/DBT than S2DM (arrows). SM: synthesized mammography; S2DM: standard two-dimensional digital mammography; DBT: digital breast tomosynthesis

**Figure 5:**
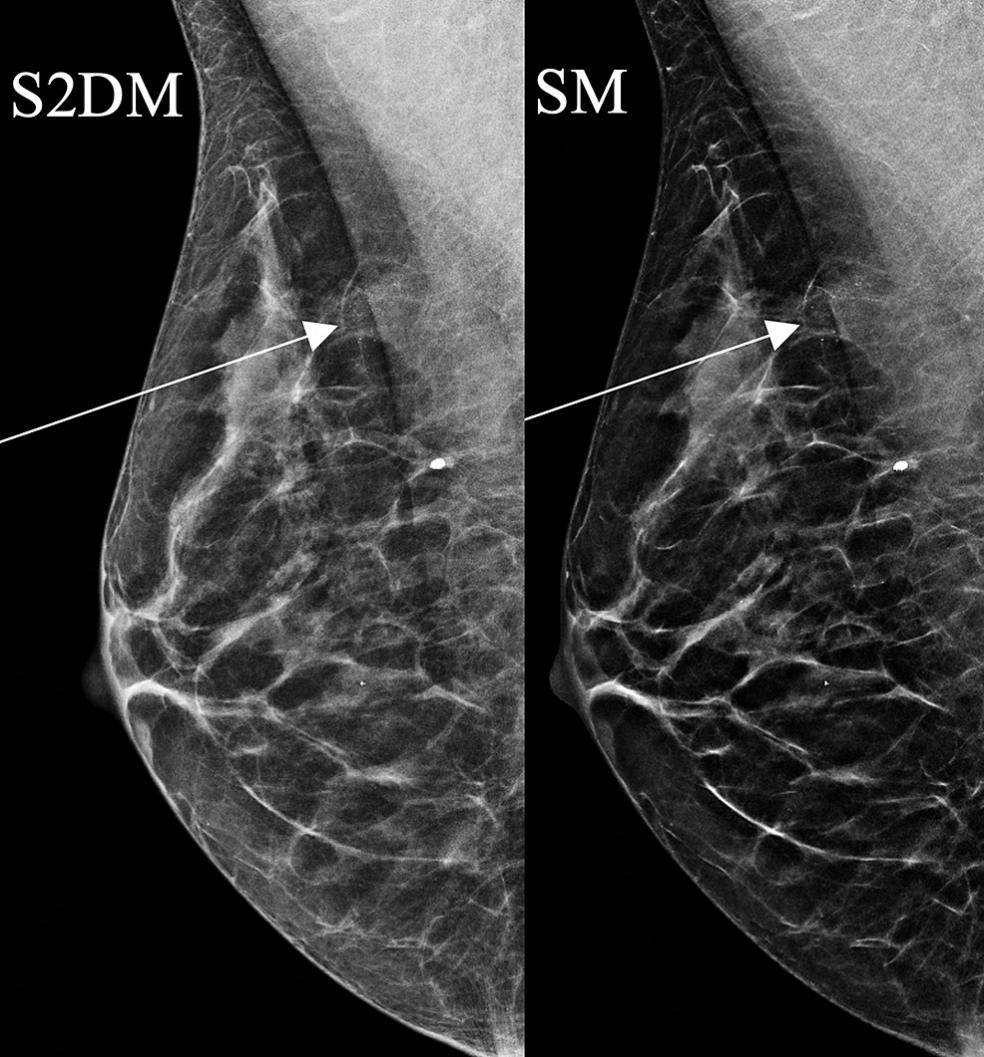
A 39-year-old woman with high-grade DCIS presented with a right breast lump. Mammography demonstrates a cluster of calcifications, which are easier to see on S2DM than SM (arrows). SM: synthesized mammography; S2DM: standard two-dimensional digital mammography; DBT: digital breast tomosynthesis; DCIS: ductal carcinoma in situ

Of note, distortion was also more easily characterized on DBT than S2DM in four (44%) of nine patients with distortion (Figure 4). Calcifications were better seen on S2DM than SM in two (5%) of 39 patients, even though the calcifications were visible on both the S2DM and the SM (Figure 5).

**Figure 6:**
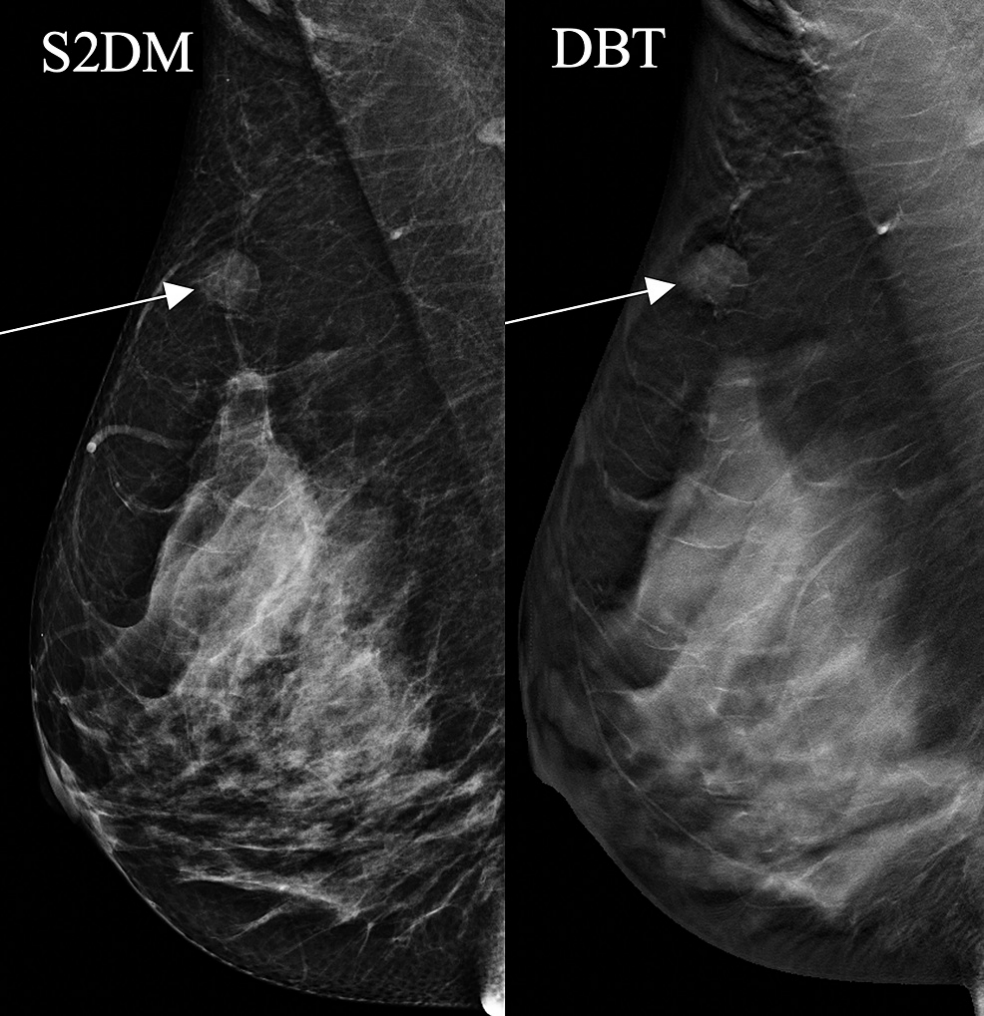
A 36-year-old woman presented with a right breast lump. Mammography demonstrated a corresponding lesion in the skin. Scrolling to the lateral most image slice on DBT confirms that the lesion is in the skin (arrows). Ultrasound demonstrated a partly intradermal lesion which likely represents an epidermal inclusion cyst. SM: synthesized mammography; S2DM: standard two-dimensional digital mammography; DBT: digital breast tomosynthesis

DBT was diagnostically helpful in numerous ways. Mass margins were better defined in 80 (71%) out of 112 patients compared with S2DM. Two masses were characterized as being intradermal on DBT (Figure 6). Multiple areas of asymmetry on S2DM images were characterized as normal glandular tissue on DBT. Distortion was seen in two patients on DBT only, but not identified on S2DM or SM. Both lesions were biopsied using tomosynthesis with benign pathology results.

Malignant lesions

Regarding the 40 patients with biopsy-proven malignancy, Table 2 lists the observed findings. All lesions were identified on S2DM, SM, and DBT. Overall, 13 patients had calcifications. These were identified on all imaging modalities, but in 11 (85%) out of 13 patients, the calcifications were more conspicuous on SM than S2DM. In 7.5% of the patients, the calcifications were easier to detect on S2DM, and in 7.5% they were equivalent. A total of 22 patients had masses. In 18 (82%) of 22 patients, DBT was better for mass margin assessment than S2DM, and in three (14%) of 22 patients, SM was better for defining mass margins than S2DM. The remainder were equivalent. Five patients had distortion. In four (80%) of 5 patients, the distortion was easier to characterize on DBT than S2DM, and in one (20%) of 5 patients, it was easier to view on SM than S2DM.

**Table 2:** Comparison of DBT and SM with S2DM in patients with malignancy. SM: synthesized mammography; S2DM: standard two-dimensional digital mammography; DBT: digital breast tomosynthesis

	DBT compared to S2DM	SM compared to S2DM
Calcifications more obvious (n = 13)	0	11 (85%)
Mass margins better defined (n = 22)	18 (78%)	3 (14%)
Architectural distortion better seen (n = 5)	4 (80%)	1 (20%)

## Discussion

While we looked at the advantages and disadvantages of DBT and SM compared to S2DM, we also looked at a direct comparison between S2DM and SM. We reviewed 400 symptomatic S2DM and SM mammograms to determine whether there was any clinical difference between these two modalities concerning detecting and characterizing various types of breast pathologies.

Our results show that the performance of SM, even without the DBT information, was not inferior to that of S2DM in terms of detectability, probability of malignancy, or lesion conspicuity. All malignant lesions were identified on S2DM, SM, and DBT. We found no clinically significant difference in image interpretation regarding the identification of asymmetries, masses, architectural distortions, or calcifications. There was no difference in the R code category assignment or breast density categorization. This indicates that S2DM alone can be used in the assessment of symptomatic breast women in a TAC, which is the current standard clinical practice. However, in patients with malignant lesions, calcifications and areas of architectural distortion were more easily identified and mass margins were more easily characterized on SM and DBT than on S2DM.

Our study has a few limitations. First, this is a single-center study and may not be representative of different populations. Because we focussed on symptomatic patients presenting to a breast TAC, we cannot comment on DBT or SM use for the interpretation of breast screening studies. Furthermore, imaging was performed with machines supplied by a single vendor (Hologic). Therefore, a multi-institutional trial with multiple radiologists with varying levels of experience and different imaging vendors is required to validate our findings. Finally, it should be emphasized that this comparison of S2DM and SM does not mean we advocate the use of SM alone. SM needs to be interpreted with the full DBT imaging dataset concerning clinical decision-making.

Although we did not include a screening population in our study, numerous studies have demonstrated significantly increased cancer detection rates, as well as lower recall rates, when introducing DBT to a screening population. A study by Gilbert et al. [17] demonstrated that DBT and SM increase sensitivity and specificity compared to S2DM alone, and would reduce the number of women recalled unnecessarily in screening cases.

In our study, two women had architectural distortion identified on DBT alone. Both underwent tomosynthesis-guided biopsy with a benign result. This highlights one of the drawbacks of using DBT in the symptomatic population. Introducing DBT will result in an increased number of benign biopsies which leads to unnecessary anxiety for the patients, and an increased workload for radiology and pathology.

It is important to note that DBT takes longer to interpret than S2DM, and therefore can have consequences on workflow in a department. Interpretation of DBT increases reading time compared to S2DM alone. A study by Zuley et al. found a 33% increase in time taken to review combined DBT plus S2DM compared to S2DM alone [18].

## Conclusions

In our study, S2DM was equivalent to DBT plus SM in the detection of malignancy. Elimination of tissue overlap in DBT also obviates the requirement for additional views if S2DM is performed alone. Therefore, this study supports the fact that DBT and SM aid S2DM in the assessment of patients presenting to symptomatic TACs. SM can provide equal and, in some cases, superior diagnostic performance with the added benefit of requiring no additional radiation exposure when synthesized from DBT data. SM images are currently not intended to be used as a stand-alone diagnostic 2D imaging modality, but should always be used in combination with the full DBT dataset. Furthermore, there is no loss of clinically relevant information by omitting S2DM for women presenting to symptomatic breast clinics and using DBT and SM alone.
